# On Hyoscyamin in Mental Diseases—Seppilli and Riva

**Published:** 1881-05

**Authors:** 


### ON HYSOCYAMIN IN MENTAL DISEASES.—SEPPILLI AND RIVA.

AFTER having exhibited the effects established by the employment of amorphous and crystallized hyoscyamin, in about forty cases of mental disease (mania acute, chronic and paroxysmal, lypemania agitata, dementia agitatas), the authors conclude that there are no good reasons for much recommending it. Leaving out curative action, which they say it does not possess, hyoscyamin, considered as a sedative or hypnotic, possesses according to their experience only two indisputable virtues, from which it is superior to chloral, and these are:

1st. The facility of its administration—made by way of hypodermic injection—without irritating the skin. They made more than four hundred hypodermic injections of hyoscyamin, and never met with any unpleasant consequences.

2d. The great promptitude and certainty of its action. In a few minutes, by its means, they have reduced at various times the agitated patients of a ward to calm and silence.

From these facts, they believe that hyoscyamin will prove of great utility in those cases in which, when it becomes imperative to remove very unquiet and noisy patients from their residence to an asylum, it is necessary to employ safe and efficacious means to calm them in the shortest possible time, so as to render their transference easier, less dangerous their control, and less necessary the use of corrective means, which, at such times, are very irritating to them. They hold it to be equally useful when the patients refuse to take chloral by the mouth, and the administraticn of glysters becomes quite impossible because of their resistance.

The dose hyoscyamin ordinarily used by them was one to five milligrammes, once daily. In some cases of chronic mania and of dementia agitata, with a view to prolong the state of calm, they administered it three times daily—at 8 A. M., 4 P. M., and 10 P. M., from 0.004 to 0.005. In two cases of chronic mania, they pushed the doses of hyoscyamin to three centigrammes daily, divided into three injections.

